# AMRFinderPlus and the Reference Gene Catalog facilitate examination of the genomic links among antimicrobial resistance, stress response, and virulence

**DOI:** 10.1038/s41598-021-91456-0

**Published:** 2021-06-16

**Authors:** Michael Feldgarden, Vyacheslav Brover, Narjol Gonzalez-Escalona, Jonathan G. Frye, Julie Haendiges, Daniel H. Haft, Maria Hoffmann, James B. Pettengill, Arjun B. Prasad, Glenn E. Tillman, Gregory H. Tyson, William Klimke

**Affiliations:** 1 National Center for Biotechnology Information, National Library of Medicine, National Institutes of Health, Bethesda, MD USA; 2 Center for Food Safety and Applied Nutrition, U.S. Food and Drug Administration, College Park, Maryland, USA; 3 Center for Veterinary Medicine, U.S. Food and Drug Administration, Laurel, MD USA; 4 Bacterial Epidemiology and Antimicrobial Resistance Research Unit, U.S. National Poultry Research Center, Agricultural Research Service, U.S. Department of Agriculture, Athens, GA USA; 5 Food Safety and Inspection Service, U.S. Department of Agriculture, Athens, GA USA

**Keywords:** Databases, Genome informatics, Antimicrobial resistance, Computational biology and bioinformatics, Microbiology

## Abstract

Antimicrobial resistance (AMR) is a significant public health threat. With the rise of affordable whole genome sequencing, in silico approaches to assessing AMR gene content can be used to detect known resistance mechanisms and potentially identify novel mechanisms. To enable accurate assessment of AMR gene content, as part of a multi-agency collaboration, NCBI developed a comprehensive AMR gene database, the Bacterial Antimicrobial Resistance Reference Gene Database and the AMR gene detection tool AMRFinder. Here, we describe the expansion of the Reference Gene Database, now called the Reference Gene Catalog, to include putative acid, biocide, metal, stress resistance genes, in addition to virulence genes and species-specific point mutations. Genes and point mutations are classified by broad functions, as well as more detailed functions. As we have expanded both the functional repertoire of identified genes and functionality, NCBI released a new version of AMRFinder, known as AMRFinderPlus. This new tool allows users the option to utilize only the core set of AMR elements, or include stress response and virulence genes, too. AMRFinderPlus can detect acquired genes and point mutations in both protein and nucleotide sequence. In addition, the evidence used to identify the gene has been expanded to include whether nucleotide or protein sequence was used, its location in the contig, and presence of an internal stop codon. These database improvements and functional expansions will enable increased precision in identifying AMR genes, linking AMR genotypes and phenotypes, and determining possible relationships between AMR, virulence, and stress response.

## Introduction

Antimicrobial resistance (AMR) is a significant public health threat, resulting in tens of thousands of deaths annually from antimicrobial resistant infections in the United States alone (https://www.cdc.gov/drugresistance/threat-report-2013/index.html). With the advent of affordable whole genome sequencing, often for surveillance purposes^1^ and as part of existing surveillance programs^2^, in silico approaches to assess AMR gene content are routinely used^3,4^. Identifying AMR gene content can lead to the discovery of novel resistance mechanisms^5^, and also can be used to predict resistance phenotypes without time-consuming phenotypic methods^6,7^.

To enable accurate assessment of AMR gene content, as part of a multi-agency collaboration, NCBI previously developed a comprehensive AMR gene database, the Bacterial Antimicrobial Resistance Reference Gene Database, and AMRFinder, an AMR gene detection tool^8^. Here, we describe the expansion of the Reference Gene Database, now called the Reference Gene Catalog, to include acid, biocide, metal, and heat resistance genes, as well as virulence genes. Users have the option to use only the core set of AMR genes, or include these genes too. The Reference Gene Catalog also contains species-specific point mutations. Genes and point mutations are classified by broad function, as well as more detailed functions, if available from the literature.

We also describe several functional expansions of AMRFinder, now incorporated into AMRFinderPlus, which include:

1. It can now use nucleotide or protein sequence, and, if both kinds of sequence are provided, can combine and reconcile the results from nucleotide and protein sequence.
2. Users have the option to run analyses on the “plus” subset of the Reference Gene Catalog. This subset includes genes related to biocide and stress resistance, general efflux, virulence, or antigenicity.
3. The functionality of AMRFinderPlus has been expanded to detect point mutations in both protein and nucleotide sequences.
4. For many genes, AMRFinderPlus now utilizes manually curated BLAST cutoffs, while maintaining the previous HMM (Hidden Markov Model) functionality.
5. Users have the option to conduct taxon-specific analyses that include, or exclude, certain genes and point mutations for multiple taxa.
6. The evidence used to identify the gene has been expanded to include whether nucleotide or protein sequence was used, its location in the contig, and possession of an internal stop codon.

These database improvements and functional expansions will enable increased precision in identifying AMR genes, linking AMR genotypes and phenotypes, and determining possible relationships between AMR genes and the critical genotypes and phenotypes of virulence and stress response.

To validate AMRFinderPlus, we have tested the tool against two different datasets, both of which consist of *Salmonella* isolates that have been typed for virulence and stress response genes^9,10^, and compared our results to those described in these earlier surveys.

## Results

### Reference gene catalog composition

In 2019, we described the first version of AMRFinderPlus (‘AMRFinder’)^8^. In the course of that work, we realized that point mutations were a critical component of resistance phenotype prediction, and so we decided to incorporate point mutation detection into AMRFinderPlus. After discussion with our collaborators who focus on the food-borne pathogens *E. coli*, *Listeria*, and *Salmonella* for both research and regulatory purposes, we chose to expand AMRFinderPlus to include identification of stress response and virulence genes to better understand the relationships between these elements and AMR genes. In light of the importance of identifying diarrheagenic *E. coli*, including Shiga toxin-producing *E. coli*, for epidemiological and regulatory purposes, initially, we have focused on identifying genes associated with diarrheagenic *E. coli*.

The Reference Gene Catalog, as of database version 2020-07-16.2, consists of 6428 genes, 627 HMMs, and 682 point mutations (https://www.ncbi.nlm.nih.gov/pathogens/refgene/). These elements are divided in 5588 AMR genes, 210 stress response genes, and 630 virulence genes (see Table 1). Among the 630 virulence genes, 117 are Shiga toxin gene variants, and 43 are intimin gene variants. Given our current focus on food-borne pathogens, most of the virulence genes are those found in *E. coli* and *Salmonella*. The stress response genes contain: 2 acid resistance genes, 52 biocide resistance genes, 8 heat resistance genes, and 148 metal resistance genes. The AMR genes (type “AMR”) contribute to resistance to 31 classes of drugs and 58 specific drug phenotypes most of which were included in the original version of AMRFinderPlus^8^, and the point mutations contribute to resistance to 25 classes of drugs and 41 specific drug phenotypes (Table S1).

**Table 1:** Current combinations of type and subtype fields in the Reference Gene Catalog.

Element type	Element subtype	Description
AMR	AMR	Antimicrobial resistance gene
AMR	POINT	Known point mutation associated with antimicrobial resistance
VIRULENCE	VIRULENCE	Virulence gene
VIRULENCE	ANTIGEN	Gene codes for a known antigen; this will be a future expansion of functionality
STRESS	ACID	Acid resistance gene
STRESS	BIOCIDE	Biocide resistance gene
STRESS	HEAT	Heat resistance gene
STRESS	METAL	Metal resistance gene

Note that “resistance” is used as a shorthand for significantly decreased susceptibility, and does not necessarily mean that the gene will confer clinical resistance.

The core genes are mostly AMR genes, with 5522 out 5588 AMR genes classified as core. The 868 plus genes cover a variety of types and subtypes (Tables S1, S2). All of the 630 virulence genes fall into the plus category.

### Validation of AMRFinderPlus

We constructed a database of AMR genes, the Reference Gene Database (https://www.ncbi.nlm.nih.gov/pathogens/refgene/). We also constructed a set of HMMs that have manually curated and validated cutoffs (https://www.ncbi.nlm.nih.gov/pathogens/hmm/), as well as a collection of point mutations. Previously, we have validated the basic AMRFinderPlus approach using a collection of over 6000 bacterial isolates^8^. As part of our validation process, we ensure we are able to detect all sequences in the Reference Gene Database as stand-alone sequences; however, there might be additional challenges that arise when we assess actual bacterial genomes due to naturally-occurring variation as well as assembly issues. Here, we describe how AMRFinderPlus performed against two different datasets that were selected due to their known AMR and stress response gene content and associated phenotypes to confirm the accuracy of its new functionality against a set of genomes, including detection of point mutations and metal resistance genes. To test these two sets, AMRFinderPlus 3.8.4 using database version 2020-07-16.2 was run on the assemblies from these studies.

### Mercury-resistant *Salmonella*

A recent study of antimicrobial-resistant *Salmonella* isolated from poultry assessed 19 resistant isolates belonging to seven serovars for antimicrobial resistance genotypes and phenotypes as well as mercury resistance genotypes and phenotypes^10^. In that analysis, both CARD^11^ and ResFinder^12^ were used to assess acquired resistance genes, so we are able to compare our results to those using different methods. For antimicrobial resistance, there were no discrepancies between AMRFinderPlus and the results of Cohen et al.^10^ for acquired genes and point mutations that confer resistance to beta-lactamases, chloramphenicol, macrolides, quinolones, sulfonamides, and tetracycline (92 presence calls and 212 absence calls). For the aminoglycoside modifying enzymes (AMEs), there were several differences. AMRFinderPlus does not report either *aac(6’)-Iy* or *aac(6’’)-Iaa*, as large-scale surveys^8,13^ indicate these do not appear to confer resistance^14^ despite their ubiquity in *Salmonella*; as a result, these are not included in the Reference Gene Catalog and would not be called by AMRFinderPlus. In addition, AMRFinderPlus called *aac(3)-Id* in strain 164132 (this is a synonym of *aacCA5*). When the same genome sequence was run through CARD’s RGI and ResFinder, both of those systems also called *aac(3)-Id* in the same location, so it is unclear why Cohen et al*.* did not identify this gene in this isolate.

Of the eight isolates that expressed a mercury resistance phenotype, AMRFinderPlus determined that each carries the *merA*, *merC*, *merD*, *merE*, *merP*, *merR*, and *merT* genes, which are found in the *mer* operon^15^, while the two mercury-sensitive phenotype isolates lacked these genes, as was found in the study.

### Assessment of plasmid gene content in multidrug-resistant IncA/C plasmids

We also examined six isolates from a comparative study of multidrug-resistant IncA/C plasmids isolated from six *Salmonella* enterica isolates, representing six different serovars^9^. Each of these isolates also had assemblies of their IncA/C plasmids closed to single circular contigs using long-read PacBio sequencing. These plasmids are a useful test set as they encode several resistance genes, including those for antimicrobial compounds, and quaternary ammonium and mercury compounds. Because these sequences are closed, we also can ensure that AMRFinderPlus is able to detect the duplicate copies of the cephalosporinase *bla*_*CMY-2*_ observed previously in several of the plasmids. In addition, we also examined the AMRFinderPlus output on the Pathogen Detection website run as part of the Pathogen Detection pipeline (https://www.ncbi.nlm.nih.gov/pathogens/microbigge/) to determine if other genes, not described by Cohen et al., were observed in the whole-genome-shotgun draft assemblies derived solely from the Illumina short-read data.

In the closed plasmid sequences, AMRFinderPlus identified the same plasmid antibiotic, quaternary ammonium, and mercury resistance genes as observed previously; in the three plasmids that have duplicate *bla*_*CMY-2*_ genes, the duplicate copies were successfully recovered. When examining the entire genome using draft assemblies generated by the Pathogen Detection system^16^, additional AMR genes were found, all of which were consistent with observed susceptibility typing. In several isolates, additional metal resistance genes were discovered. Analyzing draft assemblies demonstrated one limitation of both AMRFinderPlus and assembly-based gene detection systems in general, which is they can be only as good as the genomic data they are assessing. In the draft assemblies, we were unable to recover two copies of *bla*_*CMY-2*_, which would be expected, as draft assemblies often will be unable to resolve multiple copies of duplicated genes or genomic regions^17^. Further manual inspection of these assemblies confirmed that these draft assemblies lacked two copies of *bla*_*CMY-2*_, so this does not appear to be an AMRFinderPlus detection problem.

## Discussion

We developed and populated a highly curated database with hierarchical structure for AMR proteins, manually curated cutoffs, and associated hierarchical names. This database now also includes point mutations, stress response and virulence genes, and it contains additional descriptive fields for each gene or point mutation. AMRFinderPlus uses this AMR protein database, HMMs, a hierarchy of AMR protein families, and a custom rule set to identify AMR genes and point mutations, stress response genes, and virulence genes. In addition, AMRFinderPlus reports the evidence used to make each call, which includes length information and contig position, so that users can evaluate its strength and their confidence in the call. We vetted this new tool against two well-characterized datasets and were able to find high concordance with previous results for both AMR and stress response genes. When the 6241 isolates described by Feldgarden et al.^8^ were reanalyzed using AMRFinderPlus results from the Pathogen Detection pipeline, 314 point mutations, 14,128 virulence genes, and 39,408 stress response genes were found in these isolates, providing a large amount of additional information about these isolates.

Since our description of the earlier version of AMRFinderPlus^8^, other widely-used tools also have made improvements, such as adding AMR ECOFF (epidemiological cut-off) predictions^18^. However, many, if not most of these tools, still rely on a nucleotide database, which can lead to allele misassignments, yielding significant differences in the interpretation of resistance^8,18^. A key component of AMRFinderPlus is its curated *protein* database of acquired genes and point mutations, which can be searched to assess either protein sequences or, using translated BLAST, nucleotide sequence. This database is available as a download, but NCBI also has developed a user interface, the Reference Gene Catalog (https://www.ncbi.nlm.nih.gov/pathogens/refgene/), which allows users to search and download gene symbols, gene names, nucleotide and protein accessions, and type, subtype, class, and subclass information for every gene and point mutation in the Reference Gene Catalog.

AMRFinderPlus results are integrated into NCBI’s Pathogen Detection Project (https://www.ncbi.nlm.nih.gov/pathogens/), which rapidly clusters and identifies related pathogen genomic sequences originating in food, environmental sources, and patients^16^. For every bacterial isolate in the Pathogen Detection system, AMRFinderPlus is run, and the results are returned for public use in two different graphical interfaces. In the Isolates Browser (https://www.ncbi.nlm.nih.gov/pathogens/isolates/), a summary of AMR, stress response, and virulence genes are displayed for each isolate of interest, and also can be downloaded for further analysis. In the Pathogen Detection system’s Microbial Browser for Identification of Genetic and Genomic Elements (MicroBIGG-E; https://www.ncbi.nlm.nih.gov/pathogens/microbigge/), AMRFinderPlus results for those isolates with genomic data deposited in GenBank are displayed in a more comprehensive format resembling the AMRFinderPlus output, while including additional data such as BioSample and strain names, and isolate source. In addition, both the Isolate Browser and MicroBIGG-E allow cross-browser selection, whereby sets of isolates or genes selected in one resource or the other allows selections in the other resource, either the set of genes encoded by the isolates, or the set of isolates that encode the genes, respectively.

AMRFinderPlus data also are used outside of NCBI. The National Antimicrobial Resistance Monitoring System uses AMRFinderPlus as the identification system for its Resistome Tracker, which follows the global spread of resistance genes and point mutations in non-typhoidal *Salmonella*^19^. AMRFinderPlus also is used as part of the Microbiological Diagnostic Unit Public Health Laboratory’s pipeline for resistance element detection (https://pypi.org/project/abritamr/). Additional studies^20,21^ tracking the spread of resistance genes and elements have used AMRFinderPlus, and the Reference Gene Catalog has been used by metagenomic analysis tools to identify metal resistance genes^22^.

In our previous work, we identified the need to incorporate the detection of point mutations and of stress response and virulence genes into AMRFinder to better assess AMR phenotypes and the linkage between AMR and other critical phenotypes^8^. While this study only examined a small set of isolates, NCBI’s Pathogen Detection system currently uses AMRFinderPlus to identify these genetic elements in over 800,000 clinical and environmental bacterial isolates (https://www.ncbi.nlm.nih.gov/pathogens/), enabling the rapid identification of isolates with important AMR-related genotypes.

## Methods

### Curation of acquired stress response and virulence genes

We have expanded the Reference Gene Catalog^8^ to include genetic elements related to stress response and virulence genes; these expansions can be visualized in the Reference Gene Catalog Browser (https://www.ncbi.nlm.nih.gov/pathogens/refgene/). One reason we expanded AMRFinderPlus is to understand the linkages between AMR genes and stress response and virulence genes in food-borne pathogens; thus, the stress response and virulence genes included in the Reference Gene Catalog are composed primarily of *E. coli*-related genes derived primarily from González-Escalona et al.^23^ as well as BacMet^24^, but also have been supplemented by manual curation efforts for other taxa. Stx gene nomenclature adopts the system of Scheutz et al.^25^ and the intimin (*eae*) gene nomenclature uses existing designations in the literature^26,27^. Genes are incorporated only if there is literature supporting the function of that protein or closely related sequences that meet the identification criteria. As a major focus of our work is to improve NCBI’s Pathogen Detection system^16^, we excluded genes that belonged to organisms not deemed clinically relevant. To remove ‘housekeeping’ proteins that were universally found in one or more taxa in the Pathogen Detection system, sequences were not included if they were found at a frequency of greater than 95% in a survey of 58,531 RefSeq bacterial assemblies belonging to any of the following species: *Acinetobacter, Campylobacter, Citrobacter, Enterococcus, Enterobacter, Escherichia/Shigella, Klebsiella, Listeria, Salmonella, Staphylococcus, Pseudomonas*, and *Vibrio*. If genes of particular interest in foodborne pathogens exceeded this threshold, they were excluded in the taxa where they appear to be nearly universal (see “Identifying genomic elements” below). In addition, genes with misidentified functions, such as copper-binding proteins that use copper as a co-factor yet do not confer resistance to copper, also were excluded. As we continue to expand the database, we use similar criteria when adding genes.

### Development of BlastRules

BlastRules are genome annotation tools that, based on identity and coverage threshold criteria, determine which genes translate into proteins that meet these criteria, and then provide annotations such as gene symbol and protein product name to all matching proteins^28^. Each BlastRule relies on one or more model proteins used in BLAST^29^ searches to find homologs that meet three separate criteria: coverage of a model protein by the alignment found by BLAST, coverage of the target protein, and amino acid percent identity computed for that alignment. BlastRules work especially well in annotation pipelines when the protein families they describe are narrowly defined, so sequence similarity levels are high, the risk of a false-negative BLAST search result is vanishingly small, and the exquisite sensitivity made possible by HMMs built from multiple sequence alignments is not needed. Every BlastRule created for AMRFinderPlus is also added to the set of annotation rules used by PGAP, NCBI’s Prokaryotic Genome Annotation Pipeline^28^, which produces bacterial genome annotation that complies with requirements for submission to GenBank, an archival public sequence database that shares data with EBI and DDBJ. The rules also are used by RefSeq, NCBI’s continuously reannotated database of non-redundant reference sequences.

BlastRules are implemented slightly differently in AMRFinderPlus. In AMRFinderPlus, the complete length identity threshold is used, even if the protein is not full-length, whereas BlastRules can have distinct identity thresholds for partial proteins. In addition, for certain genes, different reference proteins or stricter cutoffs might be used in AMRFinderPlus, as some BlastRules have low identity thresholds and search for different proteins in order to identify distant homologues that might differ functionally from those genes in the Reference Gene Catalog. Thus, it is possible that PGAP annotations might diverge slightly from AMRFinderPlus results. The genes currently assessed using BlastRules are described in Table S3.

### Curation of point mutations

Point mutations were extracted from the literature as well as existing databases such as CARD^11^ and ResFinder^12^. All were assigned type AMR, and subtype POINT (Table S1). Reference sequences were vetted to ensure that all wild type point mutations, either protein for protein coding sequence or nucleotide for non-coding regions, were present in the reference sequence. In addition, only full-length protein or nucleotide sequences were used. For non-coding nucleotide sequences, the reported location of some point mutations was adjusted to match the coordinates on that species’ reference sequence, as opposed to a canonical *E. coli* sequence. Every point mutation is assigned to a taxonomic group (see Table 2 for taxonomic groups), and only will be searched for when the user indicates query sequences belong to a specific taxonomic group; point mutation identification is not a default setting. Identical point mutations belonging to different taxa are considered to be distinct elements in the Reference Gene Catalog.

**Table 2:** Taxa for which genetic elements can be excluded or included.

Organism option	Point mutation screening	Are certain plus genes excluded?	Taxa
Campylobacter	Yes	No	*Campylobacter coli* and *C. jejuni*
Enterococcus_faecalis	Yes	No	*Enterococcus faecalis*
Enterococcus_faecium	Yes	No	*Enterococcus faecium*
Escherichia	Yes	Yes	*Escherichia* sp. including *Shigella, E. albertii, E. fergusonii*
Klebsiella	No	Yes	*Klebsiella pneumoniae, K. oxytoca*
Salmonella	Yes	Yes	*Salmonella* sp.
Staphylococcus_aureus	Yes	No	*Staphylococcus aureus*
Staphylococcus_pseudintermedius	No	Yes	*Staphylococcus pseudintermedius*
Vibrio_cholerae	No	Yes	*Vibrio cholerae*

Organism option describes the value used by the  --organism flag. Taxa describes the species referred to by that flag.

### Classification of genes and point mutations

Every element (gene or point mutation) is assigned a type, subtype, class, and subclass, so users can search for functional groupings of genes and point mutations. Element type and subtype broadly define the element, as shown in Table 2, "Element type" contains three categories, AMR, STRESS, or VIRULENCE. "Element subtype" is a duplicate of "Element type" unless a more specific category has been defined.

Class and subclass provide more specificity. For AMR elements (type “AMR”, see Table S1), class describes the broad class or classes of antibiotics to which the element confers or contributes to resistance. Subclass describes particular antibiotics, but this list should not be considered exclusive. Where the literature is unclear, contradictory as to resistance phenotype, or the effect of the element is highly dependent on strain or species background, the class descriptor is used to indicate this uncertainty.

For stress response and virulence genes (types “STRESS” and “VIRULENCE” respectively), *stx* and intimin elements are assigned classes and subclasses. For *stx*, class indicates membership in the stx1 or stx2 family, while subclass indicates to which *stx* type the *stx* protein subunit belongs. For those Stx proteins lacking complete identity to sequences in the Reference Gene Catalog, the closest protein hit might not correspond with the closest *stx* nucleotide type sensu Scheutz et al.^25^, since closely related proteins do not always correspond to the closest nucleotide sequence. For intimin genes (*eae*), the class field indicates that the protein is an intimin protein, while the subclass indicates the family to which the intimin belongs.

In addition, curated genes are assigned to one of two categories, “core” or “plus.” “Core” includes AMR-specific genes and proteins from the Bacterial Antimicrobial Resistance Reference Gene Database (BioProject PRJNA313047), plus point mutations. The sources of input for this curated database include allele assignments by NCBI, exchanges with other external curated resources such as the CARD^11^, and reports of novel antimicrobial resistance proteins in the literature. Users have the option to run analyses on the “plus” subset of the Reference Gene Catalog. This subset includes genes related to biocide and stress resistance, general efflux, virulence, or antigenicity.

### Taxon-specific analyses

AMRFinderPlus has the option to exclude or include genetic elements based on taxonomic grouping (see Table 1). Using the optional—organism flag, AMRFinderPlus will automatically include taxon-specific genetic elements, such as point mutations, and exclude other genes based on taxon. Currently, the Reference Gene Catalog contains only fourteen acquired genetic elements (i.e., genes, not point mutations) excluded based on taxon. Here, the biological rationale was to identify genes which are ubiquitous in one or several taxa, and thus of little interest when found in those taxa, but should be identified in atypical taxa, as this might suggest horizontal gene transfer.

### Identifying genomic elements

AMRFinderPlus uses the database of reference sequences, HMMs, the hierarchical tree of gene families, and a set of rules to generate names and coordinates for genes, along with descriptions of the evidence used to identify the sequence. AMRFinderPlus can be run in three modes: (1) on nucleotide sequence (2) on protein sequence, or (3) on both protein and nucleotide sequence. The most accurate method is to use both nucleotide and protein sequence, in conjunction with a .gff file that provides location information. This allows HMM use (protein sequence), as well as point mutation detection in non-coding sequences and internal stop codon detection (nucleotide sequence), while enabling the removal of redundant information from the nucleotide and protein analyses.

Additional capabilities to identify point mutations and avoid reporting common genes are enabled by using the—organism option for taxa with taxon-specific information in the database. Point mutations are identified by aligning the assembled nucleotide or protein sequence to reference sequence with BLAST and assessing the amino acid(s) or nucleotide(s) at a given position in the alignment. Protein sequences and HMMs are assigned to nodes in a naming family hierarchy as described above, enabling the accurate naming and identification of both novel and known protein sequences. Links to software and documentation are available at https://www.ncbi.nlm.nih.gov/pathogens/antimicrobial-resistance/AMRFinder/ and https://github.com/ncbi/amr.

**Figure 1:**
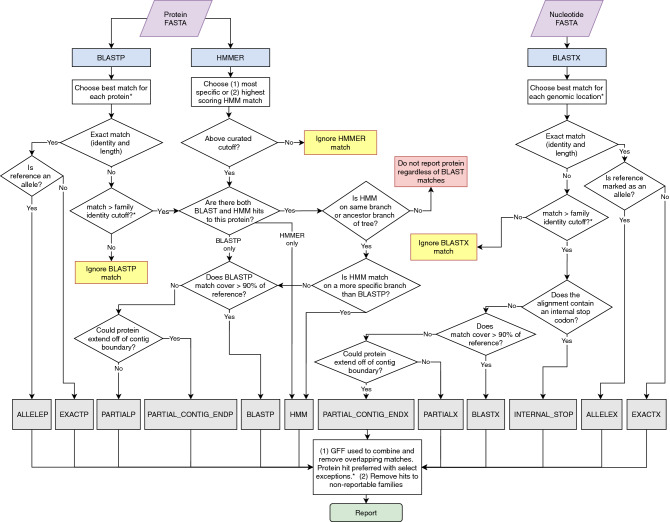
Overview of AMRFinderPlus gene identification method using protein and nucleotide sequences. When AMRFinderPlus is run in protein-only or nucleotide-only modes only the relevant side of the flowchart above is utilized. Note that HMMs are not used with nucleotide-only data which results in reduced sensitivity for some families. "Ignored" matches are not used, however an alternative method may still report the protein (e.g., an ignored HMM match will not suppress reporting based on BLASTP or BLASTX hit). *Details are simplified in this diagram; a more detailed description is included in the methods text.

Genes are reported with the following procedure after HMMER and BLAST searches are run (Fig. 1).

*Protein BLAST matches:* If protein sequences are provided as input, BLASTP^29,30^ is run with the -comp_based_stats 0 -evalue 1e-10 options against the AMR protein database described above. Matches with percent identity lower than the—ident_min parameter (default of < 90%) or, if designated, a BlastRule cutoff are dropped. “Partial” matches with percent coverage lower than the—coverage_min parameter (by default < 50% length of reference) are dropped. Partial matches to fusion proteins are dropped, as well as partial matches that cover less than 35 aa of the reference protein and which are not at the ends of contigs. From the remaining matches the sequence identifying BLAST match chosen will be the match that is (in priority order): (1) identical to a reference protein, (2) with the highest number of identical residues, or (3) the same number of identical residues, but to a shorter reference protein. In the case of a remaining tie, the match with the alphabetically smaller reference accession is chosen. If users wish to see all matches, the—report_all_equal option will report a row for each equidistant match.

*HMM matches*: To determine HMM matches, HMMER^31^ is run using the --cut_tc -Z 10,000 options with the HMM database described above. HMM matches with a full_score < TC1 and/or a domain_score < TC2 are dropped. If there are multiple HMM matches, the following criteria, in order, are used to identify the best HMM match: (1) the most specific HMM is selected (e.g., *blaKPC* is preferred to class A beta-lactamase); (2) if multiple HMM matches have the same specificity, then the HMM match with the highest full_score is preferred. Ties are broken by selecting the HMM with the highest TC1; if HMMs have the same TC1, then the first HMM, when HMM identifiers are sorted alphabetically, is chosen.

*Combining HMM and protein BLAST matches:* If a BLASTP match above cutoff is available it is used to determine the symbol/name of a protein unless there is an HMM match and the family of the HMM is not a proper descendant of the family of the reference protein. In this case the protein is not reported. A full-length BLASTP match with >  = 98% identity to a reference protein whose family has an HMM is required to have a hit to this HMM, otherwise the BLASTP match is ignored.

*Translated DNA BLAST matches:* Translated blast (BLASTX) is run using the options -comp_based_stats 0 -evalue 1e-10 -word_size 3 -seg no -max_target_seqs 10,000 -query_gencode TRANSLATION_TABLE, where TRANSLATION_TABLE is the NCBI genetic code which is 11 by default. The BLAST database and the algorithm for selecting hits are the same as described above for proteins, but note that HMM searches are not performed against the unannotated assembly. Unlike protein search, premature stop codons and frame shifts that lead to early stop codons are detected by the presence of a stop codon internal to the alignment.

*Combining protein and translated results:* Running AMRFinderPlus with both nucleotide and protein sequence allows it to correctly identify elements in spite of some errors or omissions in structural annotation by using translated BLAST results. By default, the BLASTP match is preferred, but if a BLASTP match is not identical and is covered at least 75% by a BLASTX match that is identical to a reference or has an internal stop codon, then the BLASTX match is returned.

*Identifying point mutations:* AMRFinderPlus identifies point mutations in database genes using BLASTP, BLASTX, and BLASTN. Reference sequences for protein point mutations are aligned by BLASTP or BLASTX as described above. The protein or translated blast results with the fewest differences to the reference is chosen; in the case of a tie the protein BLAST is preferred, and all else being equal, the blast match with the alphabetically smaller reference accession is chosen. Nucleotide point mutations, such as promoter or 16S mutations, are assessed by running BLASTN against reference sequences using options -evalue 1e-20 -dust no. Nucleotide alignments for point mutation detection must have a segment of at least 96% similarity and at least 401 bp in length or, if the reference sequence is shorter than 401 bp, the length of the reference, since AMRFinderPlus, when possible, uses 200 bp of flanking region surrounding the point mutation to prevent misidentification of the particular position. From these alignments, BLAST hits containing the reference allele, with the highest identity over that segment are chosen. For all point mutations, the bases adjacent to the mutation must be identical to the reference for the mutation to be reported.

*Reporting results:* Each protein family has one of three reporting statuses: non-reportable, plus-reportable or core-reportable. Plus-reportable families are reported only if the—plus option is present. Non-reportable families are used to suppress reporting of proteins in otherwise reportable clades, such as metallohydrolases that are closely related to metallo-beta-lactamases but which do not confer beta-lactamase resistance^32^. The option—report_common in combination with -o/–organism will add the reporting of common “plus” genes that are, by default, suppressed for a given organism. The option—mutation_all prints all observed and non-observed reference SNP mutations into a specified file. The type of mutation is indicated by a keyword added to the “Sequence name” column of the report: this keyword is “[WILDTYPE]” for non-observed reference alleles and “[UNKNOWN]” for observed non-reference alleles.

*AMRFinderPlus “Method”:* AMRFinderPlus hits are assigned a “method” based on the way they were detected and the characteristics with which they were detected. This helps to assess the probability that the element is functional and how it was detected. A suffix on the method of *P*, *X*, or *N* indicates that the element was detected using protein, translated nucleotide, or nucleotide BLAST respectively. *EXACT* and *ALLELE* indicate that full-length identical matches were made between the element and the reference for a gene or allele respectively. *BLAST* indicates that the element was identified by a BLAST alignment that was not identical as above, but the alignment was greater than the curated identity cutoff or 90% by default over more than 90% of the sequence. The method *PARTIAL* is returned when a blast hit of less than 90% length is internal to a contig. If the gene is < 90% of the reference length and has partiality and orientation that could allow it to run off a contig boundary, the method *PARTIAL_CONTIG_END* is returned. The method *HMM* is used for elements that do not meet BLAST thresholds, but align to HMMs above the curated cutoff. Translated sequences can be used to identify proteins with stop codons internal to the alignment to reference. Such proteins are assigned the method *INTERNAL_STOP*. Point mutations are given the method *POINT*.

*Reporting possibly non-functional protein sequences:* In the interests of sensitivity and possible epidemiological utility, AMRFinderPlus may report hits that might not confer the expected phenotype, but for each reported hit, AMRFinderPlus provides sufficient information to assess the functionality of the protein. This information includes the “method,” percent identity of the matching sequence, the proportion of the reference sequence that is covered by the match, the actual hit length, the reference protein length, and the HMM matched when an HMM hit is found. We would note, however, that AMRFinderPlus would be unable to detect phenomena such as resistance gene silencing^33^.

## Supplementary Information

Supplementary Information.

## Data Availability

The Reference Gene Catalog is available at https://www.ncbi.nlm.nih.gov/pathogens/refgene/, the Reference HMM Catalog at https://www.ncbi.nlm.nih.gov/pathogens/hmm/. AMRFinderPlus source code can be found at https://github.com/ncbi/amr/wiki.
